# Mr. Watt, on Varus and Valgus

**Published:** 1801-02

**Authors:** R. Watt

**Affiliations:** Paisley


### *To the Editors of the Medical and Phyfical Journal.*

Gentlemen,

I HATE controverfy, and therefore approve much of the *present* conduct of your correfpondent, Mr. Sheldrake, in dropping the fubject of difpute between us. It is certainly the moft moſt rational mode of procedure, when he finds his attack no longer *defensible*, to get rid of it *in the best way he can.** Whether his Anſwer, or my Reply, contains moſt *unprovoked*, *illiberal*, and *personal abuse*, I leave to your candid Readers to determine.

I hope, however, you will excuſe my taking notice of a very ſingular paſſage, principally directed againſt me,† in a latter communication from the ſame author. It is as follows.

"Gentlemen, I ſhall now, with your permiſſion, reſume the ſubject I hinted at in my laſt communication, viz. to ſhow, as far as my experience will ſhow, under what circumſtances, and at what periods of life, thoſe diſtortions of the feet, commonly called Club-feet, and more learnedly, *but more vaguely*, *termed* VARUS and VALGUS, may be cured."‡ With regard to the terms *varus* and *valgus*, I may remark, that they have been uſed not only by medical writers, but even by claſſical authors, and in common language, for at leaſt *two thousand years*, as appears from the following paſſage in Plautus: "*Aut varum aut valgum aut compernem*, *&c.* *Mil.* 3, 1, 128; and ſeveral others that I could produce, equally ancient. And during which *long* period they have been *invariably* uſed to ſignify the ſame things; *varus* when the toes are turned in, and *valgus* when they are turned out. It is certainly *one step* towards perfection in any language, when there are *two distinct species* of a thing, to have an appropriate name for each; yet it would appear, that there are ſome people ſo eccentric, or I may rather ſay, abſurd in their notions, as to *prefer one confused term*, *equally inapplicable to either species*, *to a proper and distinct name for each.* I would aſk Mr. S. what *striking resemblance* he can perceive between a Club and a Diſtorted Foot, let him take any CLUB he chuſes, from the celebrated Club of Hercules to that of a common *Cow-keeper.* I am, &c.

*Paisley, Jan.* 10, 1801. R. WATT.

*P. S.* In the courſe of a month or two, I ſhall tranſmit you two caſes of *Vari*, which I have now very nearly cured; the one a boy between two and three years of age, the other between four and five. With theſe I ſhall alſo give you a deſcription and drawing of the machine I have uſed for that purpoſe.

* Medical and Phyſical Journal, Vol. V. p. 9.

† Ibid. Vol. IV. pp. 192, 193. ‡ Ibid. Vol. IV. p. 493.

